# The association between soluble suppression of tumorigenicity-2 and long-term prognosis in patients with coronary artery disease: A meta-analysis

**DOI:** 10.1371/journal.pone.0238775

**Published:** 2020-09-04

**Authors:** Niannian Liu, Tao Hang, Xiang Gao, Wenxue Yang, Wenjie Kong, Qiaozhen Lou, Jiming Yang

**Affiliations:** 1 Department of Cardiology, the Fourth Affiliated Hospital of Nanjing Medical University, Nanjing, China; 2 Department of Cardiology, the Second Affiliated Hospital of Nanjing Medical University, Nanjing, China; Scuola Superiore Sant'Anna, ITALY

## Abstract

**Objective:**

Findings regarding the prognostic value of soluble suppression of tumorigenecity-2 (sST2) in patients with coronary artery disease (CAD) remain inconsistent. Therefore, we conducted this meta-analysis to investigate the long-term prognostic value of sST2 in patients with CAD.

**Methods:**

A comprehensive literature search was conducted across the PubMed, Embase, and Cochrane Library databases up to June 3, 2020. The primary outcome was major adverse cardiac events (MACEs). The secondary outcomes were all-cause mortality, cardiovascular (CV) death, heart failure (HF), and myocardial infarction (MI). Pooled estimations and 95% confidence intervals (CIs) were assessed using a random-effects model.

**Results:**

Twenty-two articles that enrolled a total of 17,432 patients with CAD were included in the final analysis. CAD patients in the highest categories of baseline sST2 had a significantly higher risk of MACEs (HR: 1.42, 95% CI: 1.09–1.76), all-cause mortality (HR: 2.00, 95% CI: 1.54–2.46), and CV death (HR: 1.42, 95% CI: 1.15–1.68), HF (HR: 2.41, 95% CI: 1.87–2.94), but not that of MI (HR: 1.15, 95% CI: -0.73–3.04), than those in the lowest categories. These results were consistent when baseline sST2 was presented as continuous values in one unit increments. Moreover, subgroup analysis showed that elevated baseline sST2 levels increased the long-term risk of MACEs in the acute coronary syndrome (ACS) population (HR: 1.74, 95% CI: 1.39–2.09) but only showed a trend toward higher risk of MACEs in the non-ACS population (HR: 1.09, 95% CI: 0.87–1.30).

**Conclusions:**

The findings suggest that a higher concentration of baseline sST2 is associated with a higher risk of MACEs, all-cause mortality, CV death, and HF in patients with CAD. Elevated sST2 levels could significantly predict future MACEs in the ACS population but not in the non-ACS population.

## Introduction

Coronary artery disease (CAD), including chronic coronary syndromes (CCS) and acute coronary syndromes (ACS), is one of the leading causes of disability and mortality worldwide [1]. The management of CAD with reperfusion strategies and pharmacological treatment have successfully decreased the incidence of major adverse cardiac events (MACEs) and all-cause mortality [2]. However, patients with CAD still have substantial risk of future cardiovascular events after the onset of the initial episode [3]. Hence, individualized risk prediction in patients with CAD has been applied to identify those with poor prognosis and to optimize cardiovascular disease prevention in high-risk patients [4]. In recent decades, there has been increasing interest in the emerging use of biomarkers as predictors for the prognosis of CAD [5].

Soluble suppression of tumorigenicity-2 (sST2), a member of the interleukin (IL)-1 receptor family, is a decoy receptor of IL-33. sST2 may be released by vascular endothelial and myocardial cells in response to cardiomyocyte biomechanical strain [6]. Excessive circulating sST2 may neutralize the protective effect of IL-33 [7, 8]. sST2 is a promising biomarker for heart failure (HF) compared to natriuretic peptides, especially as sST2 concentrations are less influenced by potential confounders, including age, sex, body mass index, and comorbidities [9, 10]. Moreover, the sST2/IL33 pathway is suggested to be involved in lipid metabolism, and increased IL-33 expression has been detected in human atherosclerotic plaques [11]. Since 2004, epidemiological studies have reported inconsistent results regarding the long-term prognostic value of sST2 in patients with CAD [12, 13]. Therefore, we conducted this meta-analysis to evaluate the relationship between sST2 and long-term clinical outcomes (e.g., MACEs, all-cause mortality, cardiovascular death [CV death], HF, and myocardial infarction [MI] events) in patients with CAD.

## Materials and methods

### Search strategy

The meta-analysis was conducted according to the Preferred Reporting Items for Systematic Reviews and Meta-Analyses statement [14]. A comprehensive literature search was performed across the PubMed, Embase, and Cochrane Library databases up to June 3, 2020, to identify studies that examined the association between baseline sST2 levels and long-term clinical outcomes using the following terms: ("suppression of tumorigenicity-2" OR "soluble suppression of tumorigenicity-2" OR “ST2” OR “sST2”) AND (“coronary heart disease” OR “coronary arterial disease” OR “cardiovascular diseases” OR “coronary disease” OR “myocardial infarction” OR “acute coronary syndrome” OR “stable coronary artery disease”). The detailed search strategy is presented in S1 Table. Furthermore, we also searched the list of references for all related publications to see whether there is other eligible literatures. Two researchers independently searched the database and reviewed all the retrieved papers. The disagreement was resolved by consulting a third researcher. The inclusion criteria were as follows: 1) cohort studies, including post hoc analyses of randomized clinical trials; 2) studies published in English; 3) studies in which sST2 values related to long-term (>9 months) clinical outcomes in patients with CAD were reported; and 4) studies in which hazard ratio (HR), relative risk (RR), or odds ratio (OR) estimates with the corresponding 95% confidence intervals (CI) for MACEs, all-cause mortality, CV death, HF, or MI events were reported or could be calculated. The exclusion criteria were as follows: 1) studies in which sST2 considered only as an element of a prognostic score and 2) studies with a duplicated population.

### Data extraction

The following information was extracted from each eligible study by two authors independently: surname name of the first author, publication year, diagnosis of the patients, sample size, age, number of males, cutoff point of sST2 levels, outcomes evaluated, follow-up duration, and variables adjusted in the multivariate model. When sST2 levels were presented as continuous variables, we standardized the group-level exposure estimates to single units, thereby allowing for the integration of the effects of different sST2 values in different studies.

### Definitions of outcomes and quality assessments

The primary outcome in this study was MACEs, which was defined as a composite outcome of death and other fatal or non-fatal cardiovascular events. Given the heterogeneity of MACEs definitions in the included studies, the potential cardiovascular events were distinct, such as MI, HF, stroke, rehospitalization, and revascularization, were included. The secondary outcomes were all-cause mortality, CV death, HF, and MI. A dedicated tool [15, 16] designed for prognostic study assessment was adopted to evaluate the quality of the included publications. This tool consists of five methodological domains to assess for bias: study participation, prognostic factor measurement, outcome measurement, description and measurement of confounding factors, and statistical analysis and reporting. After assessment, every methodological domain was labeled as good, adequate, or unclear for each study.

### Statistical analysis

Statistical analyses were performed using STATA software (version 12.0; StatCorp., College Station, TX, USA), *and P* < 0.05 was considered statistically significant. Multivariate adjusted HRs/RRs/ORs with 95% CI for each study were extracted and analyzed. For studies using the sST2 level as the categorical variable, the values of the effects for comparing the highest and lowest categories of sST2 level were extracted. The inverse variance-weighted mean of the logarithm of HR/RRs/ORs and 95% CI with a random effect model was used to calculate the association between sST2 levels and different clinical outcomes among CAD patients.

The *I*^*2*^ and Cochrane’s Q tests were adopted to evaluate the heterogeneity among different studies [17, 18]. Significant heterogeneity was considered if *I*^*2*^ > 50% [19]. Meta-regression was performed to explore the study and population characteristics that may contribute to heterogeneity [20]. Publication bias was evaluated by funnel plots using the Egger regression asymmetry test [21].

## Results

### Literature search and study characteristics

**Fig 1:**
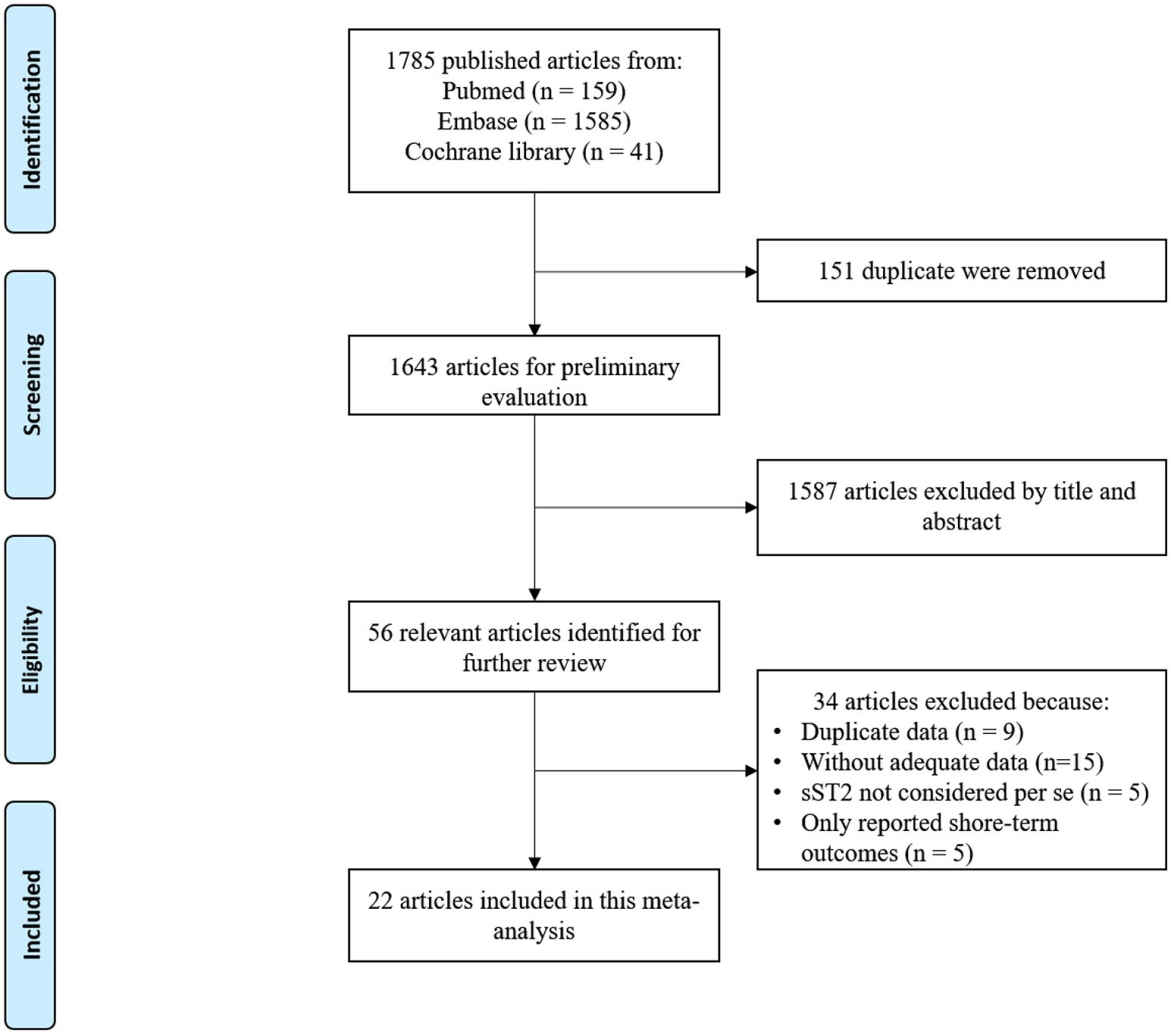
Flow diagram of screened, excluded, and analyzed studies.

The literature search process is summarized in Fig 1. We identified 159 articles from PubMed, 1,585 from Embase, and 41 from the Cochrane Library. Among them, 22 articles that enrolled a total of 17,432 patients with CAD investigated the relationship between baseline sST2 levels and long-term clinical outcomes in patients with CAD were included [12, 13, 22–41]. In these studies, the baseline sST2 level was provided as a categorical variable (18 articles) or a continuous variable (8 articles). The main characteristics of these studies are summarized in Tables 1 and 2. Regarding the types of CAD, 17 studies were conducted on the ACS population and 5 were conducted on the non-ACS population. MACEs as endpoints were reported in 13 articles, followed by all-cause mortality in 12, CV death in 5, HF in 4 articles, and MI in 2. The duration of follow-up in the included studies varied from 9 months to 13 years. Various confounding factors such as age, sex, medical history, comorbidities, biochemical parameters, and medication were adjusted when presenting the HRs/RRs/ORs in the included studies.

**Table 1:** Characteristics of studies on long-term prognosis value of baseline sST2 as categorical variable in patients with CAD.

Study	Country	Study design	Types of CAD	Patients, n	Age, Mean/Median, years	Males, n	Cut-off value	Endpoints	Follow-up duration	Variables in multivariate model
Dhillon 2011 [22]	UK	Retrospective cohort	NSTEMI	577	70 ± 13	397	638 pg/mL	MACEs (defined as the composite of all-cause mortality, HF hospitalization, and reinfarction), All-cause mortality, HF, MI	532 days (range 150–1059 days)	age, gender, previous angina or AMI, HF, HT, DM, Killip class, eGFR, log10 glucose level, therapy with β-blockers, or statins. log10 troponin I, previous history of hypercholesterolemia, or smoking
Kohli 2012 [23]	Multinational	RCT post-hoc	NSTEMI	4426	NA	2862	24.2 ug/mL	MACEs (defined as the composite of cardiovascular death and new or worsening HF), All-cause mortality, CV death, HF	1 year	TIMI risk score smoking, history of HF, creatinine clearance<60mL/min, cTnI, BNP, myeloperoxidase (MPO), and high-sensitivity C-reactive protein (hsCRP).
Dhillon 2013 [24]	UK	Retrospective cohort	Unselected STEMI	667	64.0 ± 12.2	505	677 pg/mL	All-cause mortality, HF, MI	median 587 days (range 134–2818 days)	age, gender, previous history of angina/AMI, HT, DM, Killip Class, eGFR, peak creatinine phosphokinase level, treatment with thrombolysis, betablockers, statins, ACE inhibitors or ARBs
Demyanets 2014 [25]	Austria	Retrospective cohort	CAD including stable angina, NSTEMI, STEMI	373	64.4 ± 12.1	247	169 pg/mL for SA, 453 pg/mL for STEMI, and 269 pg/mL for NSTEMI	All-cause mortality	mean 43 months	age, sex, HT, smoker, hyperlipidaemia, DM, BMI, serum creatinine
Dieplinger 2014 [26]	Germany	Prospective cohort	Stable CAD	1345	65 (58–71)	1008	19.4 ng/mL	CV death	a median of 9.8 years	sex, age, BMI, HT, dyslipidemia, DM, smoking, prior MI, peripheral artery disease, cerebrovascular disease, resting heart rate, LVEF, eGFR, Cholinesterase, hs-CRP, IL-6, NT-proBNP, hs-cTnT, Galectin-3
Minamisawa 2016 [27]	Japan	Prospective cohort	Prior MI	430	66 ± 12	343	NA	MACEs (defined as the composite of all-cause death, MI, stroke, or hospitalization due to congestive HF)	3 years	age, chronic kidney disease, BNP, GDF-15
Jenkins 2017 [28]	US	Prospective cohort	Prior MI	1401	67.3 ± 14.9	854	The highest tertile versus lowest tertile	All-cause mortality, HF	3.6 ± 1.8 years	age, sex, Charlson comorbidity index, Killip class, and maximum troponin T
Pfetsch 2017 [29]	Germany	Prospective cohort	Stable CAD	1081	58.9 ± 8.0	915	The highest quartile versus lowest quartile	MACEs (defined as the composite of fatal and non-fatal cardiovascular events), All-cause mortality, CV death	13 years	age, gender, school education, rehabilitation clinic, smoking status, history of DM, PCI, left ventricular function, HDL-cholesterol, LDL-cholesterol
Yu 2017 [30]	South Korea	Prospective cohort	STEMI undergoing PCI	323	59.1±13.1	272	75.8 ng/mL	MACEs (defined as the composite of cardiovascular death, non-fatal MI, non-fatal stroke, and ischemia-driven revascularization)	1 year	age, LVEF, final TIMI flow grade, NT-proBNP, troponin I, hs-CRP, glucose level
Liu 2018 [31]	China	Prospective cohort	STEMI receiving PCI	305	NA	243	32.2 ng/mL	MACEs (defined as the composite of all-cause death, heart failure, and nonfatal myocardial infarction), All-cause mortality	1 year	age, sex, time from onset to ER, Killip class, LVEF, NT-proBNP, cTnI-peak, hs-CRP
Lepojarvi 2018 [32]	Finland	Prospective cohort	Stable CAD	1946	NA	NA	21.5 ng/mL	All-cause mortality	76 ± 20 months	age, sex, BMI, CCS grading of angina pectoris, LVEF, eGFR, albumin-creatinine-ratio, glycated hemoglobin, and diabetes
Huang 2018 [33]	China	Prospective cohort	Unselected STEMI	186	68.5 (32–72)	119	56 ng/mL	MACEs (defined as the composite of cardiovascular death, worsening HF and recurrent MI)	1 year	SBP, Log NT-proBNP, Final low BB status
Heydari 2018 [34]	US	RCT post-hoc	AMI	317	NA	NA	35 ng/mL	MACEs (defined as the composite of cardiovascular death and hospitalization for ADHF)	3 years	baseline and cardiac magnetic resonance variables
Jha 2018 [35]	India	Prospective cohort	ACS	122	NA	90	36.5 ng/mL	MACEs (defined as the composite of unstable angina/NSTEMI, stroke, recurrent MI, re-hospitalization, and death)	9 months	age, gender, types of ACS, LVEF, DM, HT, and eGFR
Jacobs 2018 [36]	US	Prospective cohort	CAD receiving CABG	1047	NA	801	3.8 ng/mL	MACEs (defined as the composite of mortality or readmission)	1 year	The Society of Thoracic Surgeons 30-day readmission model
Zagidullin 2020 [13]	Russia	Prospective cohort	STEMI recerving PCI	156	NA	118	27.2 ng/mL	CV death	734.2 ± 61.2 days	Age, gender, hs-Troponin, LVEF
Kim 2020 [12]	South Korea	Retrospective cohort	STEMI recerving PCI	184	61.4 ± 11.8	156	The highest tertile versus lowest tertile	MACEs (defined as the composite of cardiovascular death, non-fatal MI, and non-fatal stroke)	1 year	SBP, symptom to door time, TIMI risk score, and CACS score
Somuncu 2020 [37]	Turkey	Prospective cohort	STEMI recerving PCI	380	NA	279	35 ng/mL	CV death	1 year	age, DM, IRA, Killip >1, SBP, LVEF

Data was presented as n, mean with SD or median with IQR.

Abbreviations: CAD: coronary artery disease; STEMI: ST-segment elevation myocardial infarction; MACEs: major cardiac adverse events; PCI: percutaneous coronary intervention; MI: Myocardial infarction; NSTEMI: non-ST elevation myocardial infarction; LVEF: left ventricular ejection fraction; hs-CRP: High sensitivity C protein; BMI: body mass index; eGFR: estimated glomerulus filtration rate; DM: diabetes mellitus: HT: hypertension; HF: Heart failure; TIMI: Thrombolysis in myocardial infarction; NT-proBNP: N-terminal pro-B-type natriuretic peptide; hs-cTnT: high-sensitivity cardiac troponin T; AMI: acute myocardial infarction; ADHF: acute decompensated heart failure; SBP: systolic blood pressure; CCS: Canadian cardiovascular society; BB: Beta blocker.

**Table 2:** Characteristics of studies on long-term prognosis value of baseline sST2 as continuous variable in patients with CAD.

Study	Country	Study design	Types of CAD	Patients, n	Age, Mean/Median, years	Males, n	Cut-off value	Endpoints	Follow-up, months or yeas	Variables in multivariate model
Eggers 2010 [38]	Multinational	RCT post-hoc	NSTEMI	403	69 (59–77)	261	per 1-SD increase	All-cause mortality	1 year	age, gender, HT, congestive HF, DM, hypercholesterolemia, smoking, previous MI, previous stroke, previous coronary revascularization, cTnT, NT-proBNP, CRP, IL-6, fibrinogen, and eGFR
Dieplinger 2014 [26]	Germany	Prospective cohort	Stable CAD	1345	65 (58–71)	1008	per 1-SD increase	CV death	a median of 9.8 years	sex, age, BMI, HT, dyslipidemia, DM, smoking, prior MI, peripheral artery disease, cerebrovascular disease, resting heart rate, LVEF, eGFR, Cholinesterase, hs-CRP, IL-6, NT-proBNP, hs-cTnT, Galectin-3
Pfetsch 2017 [29]	Germany	Prospective cohort	Stable CAD	1081	58.9 ± 8.0	915	per 1 log-unit increase	MACEs (defined as the composite of fatal and non-fatal cardiovascular events), All-cause mortality, CV death	13 years	age, gender, school education, rehabilitation clinic, smoking status, history of DM, PCI, left ventricular function, HDLcholesterol, LDL-cholesterol
Wang 2017 [39]	China	Retrospective cohort	AMI receiving PCI	180	61.4 ± 8.9	110	per 1 unit increase	MACEs (defined as the composite of all-cause death, stent thrombosis, myocardial infarction and target vessel revascularization)	1 year	IL-33, BNP, Gensini score, hs-CRP
Tyminska 2017 [40]	Poland	Prospective cohort	STEMI recerving PCI	117	61 (50.7–67)	82	per 10 units increase	MACEs (defined as the composite of all-cause death, HF, and non-fatal AMI)	1 year	age and NT-proBNP
Liu 2018 [31]	China	Prospective cohort	STEMI receiving PCI	305	NA	243	per 1 unit increase	MACEs (defined as the composite of all-cause death, heart failure, and nonfatal myocardial infarction), All-cause mortality	1 year	age, sex, time from onset to ER, Killip class, LVEF, NT-proBNP, cTnI-peak, hs-CRP
Gerber 2018 [41]	US	Prospective cohort	Prior MI	1401	67.3 ± 15	853	per 1 log-unit increase	All-cause mortality	1 year	GRACE score and Charlson comorbidity index
Jacobs 2018 [36]	US	Prospective cohort	CAD receiving CABG	1047	NA	801	per 1 log-unit increase	MACEs (defined as the composite of mortality or readmission)	1 year	The Society of Thoracic Surgeons 30-day readmission model

Data was presented as n, mean with SD or median with IQR. Abbreviations as in Table 1.

### sST2 and long-term clinical outcomes in patients with CAD

**Fig 2:**
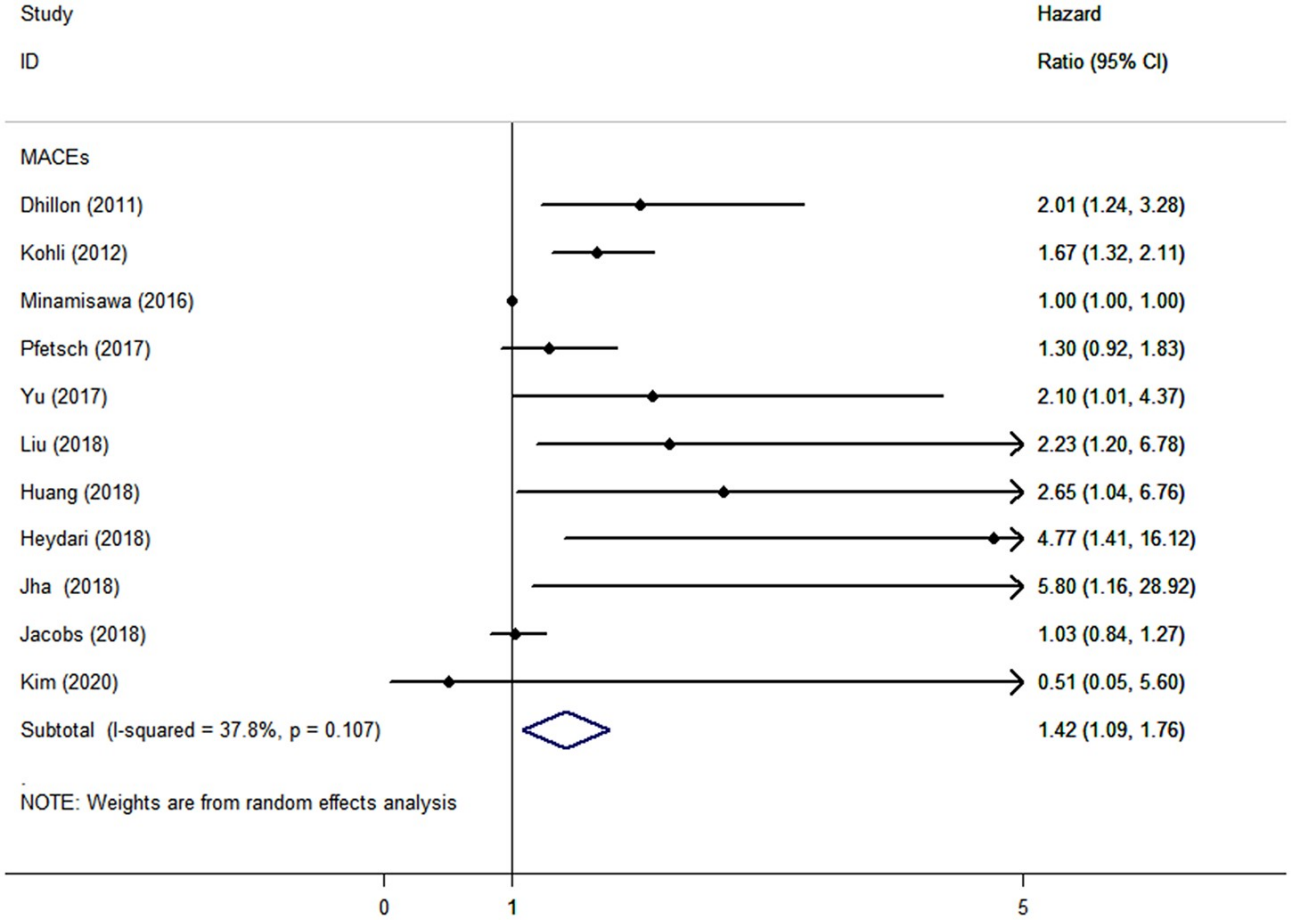
The forest plot between sST2 level as categorical variable and primary outcome in patients with CAD.

**Fig 3:**
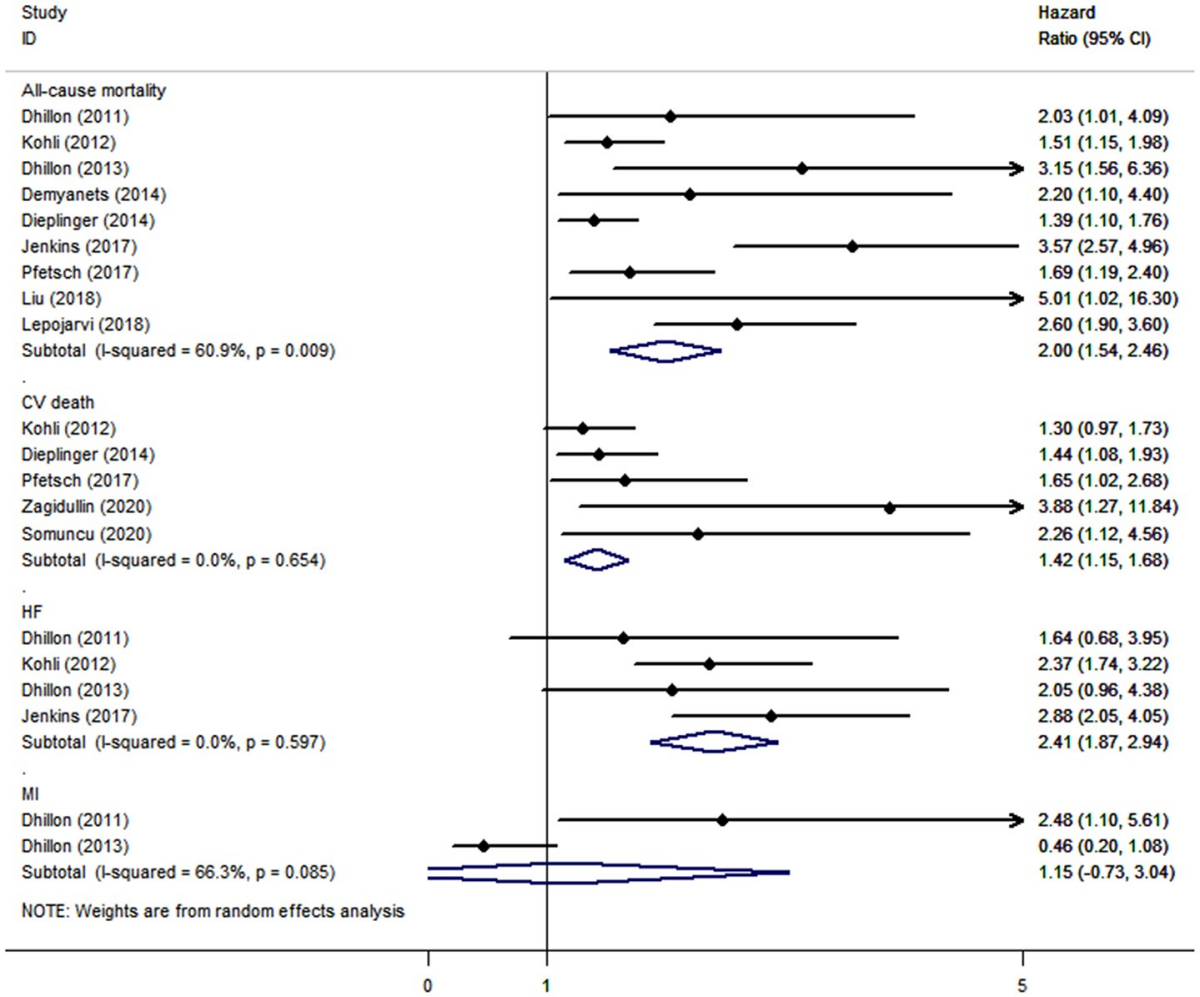
The forest plot between sST2 level as continuous variable and primary outcome in patients with CAD.

**Fig 4:**
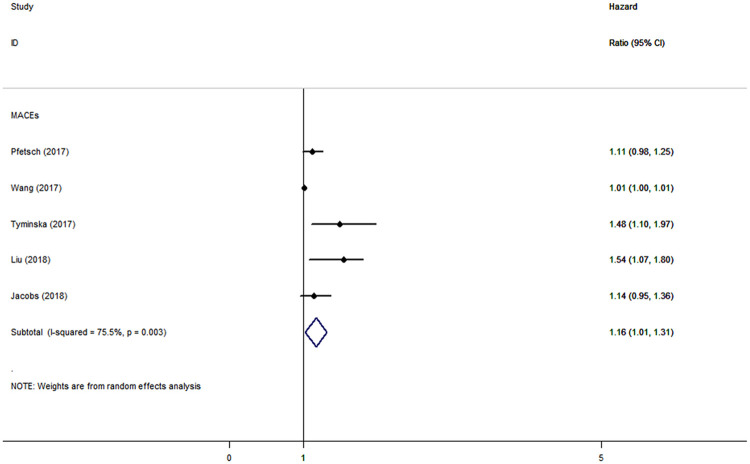
The forest plot between sST2 level as categorical variable and secondary outcomes in patients with CAD.

**Fig 5:**
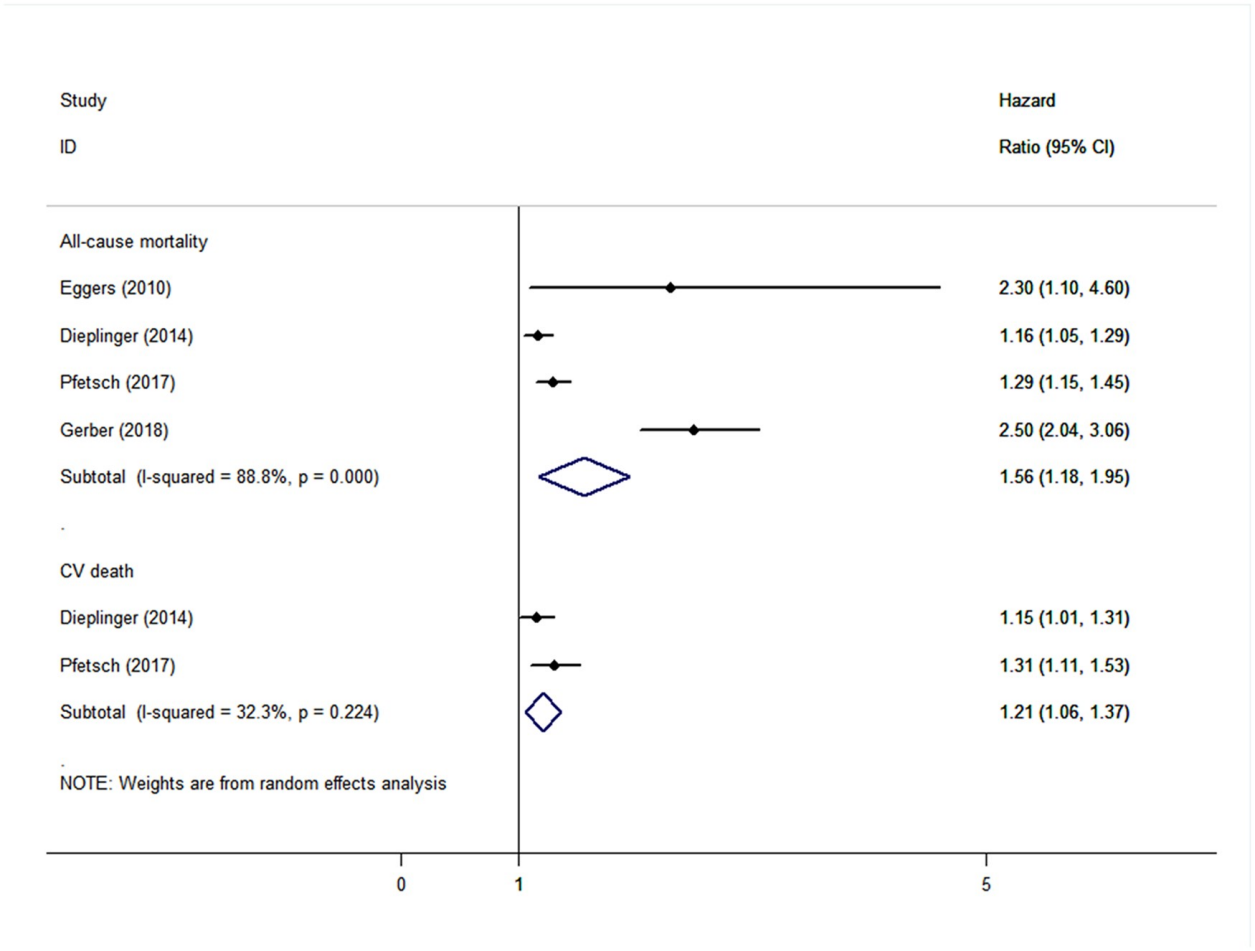
The forest plot between sST2 level as continuous variable and secondary outcomes in patients with CAD.

CAD patients in the highest categories of baseline sST2 had significantly higher risk of MACEs (HR: 1.42, 95% CI: 1.09–1.76, *I*^*2*^ = 37.8%) (Fig 2), all-cause mortality (HR: 2.00, 95% CI: 1.54–2.46, *I*^*2*^ = 60.9%), and CV death (HR: 1.42, 95% CI: 1.15–1.68, *I*^*2*^ = 0), HF (HR: 2.41, 95% CI: 1.87–2.94, *I*^*2*^ = 0), but not of MI (HR: 1.15, 95% CI: -0.73–3.04, *I*^*2*^ = 66.3%), than those in the lowest categories (Fig 3). The results were consistent when baseline sST2 was presented as continuous with 1-unit increments, with HRs of 1.16 (95% CI: 1.01–1.31, *I*^*2*^ = 75.5%) in MACEs (Fig 4), 1.56 (95% CI: 1.18–1.95, *I*^*2*^ = 88.8%) in all-cause mortality, and 1.21 (95% CI: 1.06–1.37, *I*^*2*^ = 32.3%) in CV death (Fig 5).

### Source of heterogeneity and subgroup analysis

There was medium (*I*^*2*^ = 37.8%) and substantial (*I*^*2*^ = 75.5%) heterogeneity for the association of sST2 with the primary outcome when sST2 was used as a categorical variable and a continuous variable, respectively. We conducted meta-regression with geographic locations, number of participants, study design, or types of CAD to investigate the potential sources of heterogeneity. The results suggested that the type of CAD might be the main source of heterogeneity (*P* = 0.009 for CAD patients with sST2 as categorical variables; *P* = 0.574 for those with sST2 as continuous variables) (S1 Fig). The subgroup analysis stratified by type of CAD is summarized in Table 3. For the primary outcome, the bias risks were similar in the ACS group (HR: 1.74, 95% CI: 1.39–2.09, *I*^*2*^ = 0; S2 Fig) and the non-ACS group (HR: 1.09, 95% CI: 0.87–1.30, *I*^*2*^ = 9.6%; S2 Fig) when sST2 was used as a categorical variable. However, the effects in the non-ACS group (HR: 1.12, 95% CI: 1.01–1.23, *I*^*2*^ = 0) was more robust than that in the ACS group (HR: 1.31, 95% CI: 0.89–1.72, *I*^*2*^ = 84.2%) when sST2 was used as a continuous variable.

**Table 3:** Subgroup analysis stratified by types of CAD.

Outcomes	ACS group		Non-ACS group	
	HR (95% CI)	*I*^*2*^/%	HR (95% CI)	*I*^*2*^/%
**Categorical variable**	(n = 6870)		(n = 2128)	
MACEs	1.74 (1.39–2.09)	0	1.09 (0.87–1.30)	9.6
All-cause mortality	2.48 (1.32–3.63)	67.4	1.82 (1.27–2.37)	59.8
CV death	1.37 (0.95–1.79)	1.6	1.48 (1.11–1.86)	0
HF	2.41 (1.87–2.94)	0	-	-
MI	1.16 (-0.73–3.04)	66.3	-	-
**Continuous variable**	(n = 602)		(n = 2128)	
MACEs	1.31 (0.89–1.72)	84.2	1.12 (1.01–1.23)	0
All-cause mortality	2.48 (1.99–2.97)	0	1.22 (1.09–1.34)	43.2
CV death	1.21 (1.06–1.37)	32.2	-	-
HF	-	-	-	-
MI	-	-	-	-

### Quality assessment

An overview of the methodological quality of all included studies is presented in S2 Table. Nine studies [12, 13, 23, 25, 29, 30, 32, 34, 38, 39] did not report well-defined details of the methods and results in the follow-up, which might have contributed to possible bias in their outcomes. Additionally, one [30] study had a high risk of population selection and another [38] did not provide definitions of the outcomes. All studies provided adequate descriptions of sST2 measurement, control of confounding factors, and statistical analysis.

### Sensitivity analysis and publication bias

Sensitivity analysis showed that no single study had a significant effect on the pooled effect, indicating that our results were statistically robust. Based on the results of Egger's test, when Sst2 was used as a categorization variable, there was no significant publication bias for the primary outcome (*P* = 0.059). However, a combination of visual assessment of funnel plots (S2 Fig) and Egger’s test provided statistical evidence of publication bias for MACEs when sST2 was expressed as a continuous variable (*P* = 0.015). This may indicate that the positive results in CAD patients with sST2 as a continuous variable might be overrepresented, potentially leading to a relative overestimation of the pooled HR in the meta-analysis.

## Discussion

In this study, elevated baseline sST2 levels in patients with CAD were positively associated with increased long-term risk of MACEs, all-cause mortality, CV death, and HF, but not of MI events. Moreover, subgroup analysis showed that the predictive role of sST2 for long-term prognosis in the ACS population was consistent with the main results, while the prognostic value of sST2 was not verified in the non-ACS population. This study is the largest meta-analysis to assess the relationship between sST2 and long-term prognosis in patients with CAD, which allows a much greater possibility of reaching reliable conclusions.

sST2 is a novel biomarker in inflammatory conditions and cardiovascular disease [6]. Over the past decade, several meta-analyses have suggested that sST2 is a prognostic indicator of aortic valve replacement [42], acute HF [9], chronic HF [10], and pulmonary hypertension [43]. Gu et al. also demonstrated that a higher circulating baseline sST2 level could predict poor clinical outcomes in patients with ACS in a meta-analysis that included 12 studies [44]. Our updated results with 17 articles involving patients with ACS are consistent with the findings of the above study, which confirmed the positive association between sST2 and increased risk of adverse events in the long-term prognosis of patients with ACS. Nonetheless, the study conducted by Gu et al. may have classified CV death events as all-cause mortality in some studies, which might bias their estimations [23, 26, 29]. Moreover, our study adds new evidence for the prognostic value of sST2 in the non-ACS population. The elevated baseline sST2 levels showed a trend toward an increased long-term risk of MACEs, all-cause mortality, and CV death in these patients, although without statistical significance.

Heterogeneity is common in meta-analyses. Therefore, it is an essential component of a meta-analysis to explore the sources of heterogeneity. In this study, we used meta-regression to explore the sources of heterogeneity. When sST2 was expressed as a categorical variable, the results showed that the type of CAD (ACS or non-ACS) might be the main source of heterogeneity for the primary outcome. In the subsequent subgroup analysis, the bias risks were similar in the ACS and non-ACS groups. Additionally, significant publication bias was detected in studies using sST2 as a continuous variable. This result might be ascribed to the fact that relatively few studies adopted continuous sST2 values and thus did not report potentially negative results.

The biological mechanism underlying the relationship between sST2 and clinical outcomes in patients with CAD is not fully understood. One plausible pathway is that sST2 leads to adverse clinical outcomes by counteracting the protective effect of IL-33. ST2 is expressed by cardiac myocytes in two forms: the soluble subtype (sST2) and the membrane-bound subtype (ST2L) [45]. Its ligand, IL-33, which is released by cardiac cells, normally protects the myocardium under pressure overload [46]. sST2 neutralizes this protective mechanism by acting as a "decoy receptor" for IL-33. When bound with sST2, IL-33 can no longer interact with membrane-ST2L to prevent the activation of downstream signaling pathways, leading to cardiomyocyte hypertrophy and remodeling. The indirect resistance to IL-33 might activate adverse cardiac hypertrophy and remodeling through lymphocyte function [47]. Another possible mechanism linking the ST2/IL-33 axis to CAD outcomes is its correlation with atherosclerosis progression and plaque destabilization [48]. It has been speculated that IL-33 reduces the risk of plaque rupture by inhibiting the immune response involved in atherosclerosis, an effect that can be reversed by sST2. Additionally, the IL-33/ST2 system might be a potential pathophysiological mediator in the inflammatory and fibrotic response to tissue injury and has been identified in inflammatory conditions and fibroproliferative diseases [11]. Accordingly, elevated serum sST2 levels in patients with CAD suggest the presence of active inflammation and fibrosis, factors that have been associated with poor prognosis [49, 50]. More importantly, the differential predictive value of sST2 on the occurrence of HF and MI in patients with CAD further suggests that sST2 is predominantly involved in the pathophysiology of HF and independent of CAD progression per se.

Our study has important strengths. First, multivariate-adjusted results were available from all included studies, reducing the likelihood of confounders. Second, all results were derived from cohorts or randomized controlled studies, which would enable us to detect the temporal relationship between sST2 and prognosis in patients with CAD. Nevertheless, several limitations of this study should be acknowledged. First, the heterogeneous definitions of MACEs in different studies might have contributed to potential bias. Second, some degree of heterogeneity was observed, which could be due to different types of CAD. Third, the use of different cutoff values for sST2 may lead to different estimated effects. However, due to the limited information available, we could not address the optimum cutoff value of sST2 for the prognosis of patients with CAD. Further studies with larger sample sizes are warranted to address this issue.

## Conclusions

This meta-analysis suggests that elevated baseline sST2 level is associated with a higher risk of MACEs, all-cause mortality, CV death, and HF in patients with CAD. Elevated sST2 levels could significantly predict future MACEs in the ACS population but not in the non-ACS population.

## Supporting information

S1 Table Keywords and search strategy in the umbrella review. (DOCX)

S2 Table Quality assessment of studies included in the meta-analysis. (DOCX)

S1 Fig Results of meta-regression analysis for MACEs among ACS and non-ACS in patients with CAD. (DOCX)

S2 Fig Funnel plot for publication bias for primary outcome in patients with CAD. (DOCX)

S1 Checklist PRISMA 2009 checklist. (DOC)
