# Supplementary material for: The association between soluble suppression of tumorigenicity-2 and long-term prognosis in patients with coronary artery disease: A meta-analysis
Source: PLoS One. 2020 Sep 4;15(9):e0238775. doi: 10.1371/journal.pone.0238775 (PMC7473587; doi:10.1371/journal.pone.0238775)
Supplement: S1 Table — (DOCX) [file pone.0238775.s001.docx]

**S1 Table. Keywords and search strategy in the umbrella review.**

| **Database** | **Keywords** |
| --- | --- |
| MEDLINE (OvidSP)  EMBASE (OvidSP)  Cochrane library (OvidSP) | 1. suppression of tumorigenecity-2  2. soluble suppression of tumorigenicity-2  3. ST2  4. sST2  5. 1 OR 2 OR 3 OR 4  6. coronary heart disease  7. coronary arterial disease  8. cardiovascular diseases  9. coronary disease  10. myocardial infarction  11. acute coronary syndrome  12. stable coronary artery disease  13. 6 OR 7 OR 8 OR 9 OR 10 OR 11 OR 12  14. 5 AND 13 |
