# Supplementary material for: The association between soluble suppression of tumorigenicity-2 and long-term prognosis in patients with coronary artery disease: A meta-analysis
Source: PLoS One. 2020 Sep 4;15(9):e0238775. doi: 10.1371/journal.pone.0238775 (PMC7473587; doi:10.1371/journal.pone.0238775)
Supplement: S2 Table — (DOCX) [file pone.0238775.s002.docx]

**S2 Table. Quality assessment of studies included in the meta-analysis.**

| Study | Study Participation | Follow-up Described | Measurement | Outcome Defined  and Described | Control of  Confounding | Analysis Described |
| --- | --- | --- | --- | --- | --- | --- |
| Dhillon 2011[22] | Good | Adequate | Good | Good | Adequate | Good |
| Kohli 2012[23] | Good | Unclear | Good | Good | Adequate | Good |
| Dhillon 2013[24] | Good | Adequate | Good | Good | Adequate | Good |
| Demyanets 2014[25] | Adequate | Unclear | Good | Adequate | Adequate | Good |
| Dieplinger 2014[26] | Good | Good | Good | Good | Adequate | Good |
| Minamisawa 2016[27] | Good | Good | Good | Good | Adequate | Good |
| Jenkins 2017[28] | Adequate | Adequate | Good | Adequate | Adequate | Good |
| Pfetsch 2017[29] | Adequate | Unclear | Good | Adequate | Adequate | Good |
| Yu 2017[30] | Unclear | Unclear | Good | Adequate | Adequate | Adequate |
| Liu 2018[31] | Good | Good | Good | Good | Adequate | Good |
| Lepojarvi 2018[32] | Good | Unclear | Good | Adequate | Adequate | Good |
| Huang 2018[33] | Good | Good | Good | Good | Adequate | Good |
| Heydari 2018[34] | Good | Unclear | Unclear | Adequate | Unclear | Good |
| Jha 2018[35] | Good | Adequate | Good | Good | Adequate | Good |
| Jacobs 2018[36] | Good | Good | Good | Good | Adequate | Good |
| Zagidullin 2020[13] | Good | Unclear | Good | Adequate | Adequate | Good |
| Kim 2020[12] | Good | Unclear | Good | Adequate | Adequate | Good |
| Somuncu 2020[37] | Good | Good | Good | Good | Adequate | Good |
| Eggers 2010[38] | Good | Unclear | Good | unclear | Adequate | Good |
| Wang 2017[39] | Good | Unclear | Good | Adequate | Adequate | Adequate |
| Tyminska 2017[40] | Good | Adequate | Good | Adequate | Adequate | Adequate |
| Gerber 2018[41] | Good | Good | Good | Good | Adequate | Good |
