# Supplementary material for: The association between soluble suppression of tumorigenicity-2 and long-term prognosis in patients with coronary artery disease: A meta-analysis
Source: PLoS One. 2020 Sep 4;15(9):e0238775. doi: 10.1371/journal.pone.0238775 (PMC7473587; doi:10.1371/journal.pone.0238775)
Supplement: S2 Fig — (DOCX) [file pone.0238775.s004.docx]

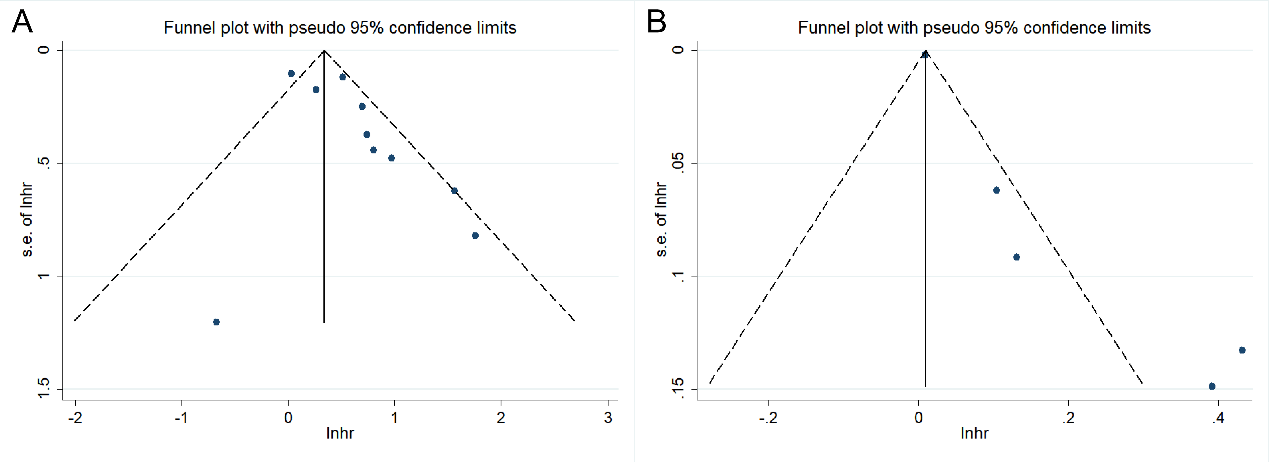


**S2 Fig. Funnel plot for publication bias for primary outcome in patients with CAD.** (A: sST2 as categorical variable, B: sST2 as continuous variable).
